# Supplementary material for: Efficient Removal of Hexavalent Chromium (Cr(VI)) from Wastewater Using Amide-Modified Biochar
Source: Molecules. 2023 Jun 30;28(13):5146. doi: 10.3390/molecules28135146 (PMC10343244; doi:10.3390/molecules28135146)
Supplement: Supplementary file 1 [file molecules-28-05146-s001.zip › molecules-2425489-supplementary.pdf]

Supplementary Materials

Efficient removal of Cr (VI) from wastewater using amide-modified biochar

Ashraf Ali [1,*], Sarah Alharthi [2,3], Nora Hamad Al-Shaalan [4], Alia Naz [5] and Hua-Jun Shawn Fan [6]

[1] Department of Chemistry, Faculty of Physical & Applied Sciences, The University of Haripur, 22620 Pakistan
[2] Center of Advanced Research in Science and Technology, Taif University, P.O. Box 11099, Taif 21944, Saudi Arabia
[3] Department of Chemistry, College of Science, Taif University, P.O. Box 11099, Taif 21944, Saudi Arabia
[4] Department of Chemistry, College of Science, Princess Nourah bint Abdulrahman University, P.O. Box 84428, Riyadh 11671, Saudi Arabia
[5] Department of Environmental Science, Faculty of Physical & Applied Sciences, The University of Haripur, 22620 Pakistan; aliaawkum@gmail.com
[6] College of Chemical Engineering, Sichuan University of Science and Engineering, Zigong city, Sichuan Province, PR China
* Correspondence: ashraf.ali@uoh.edu.pk; Tel.: (+923471214422)

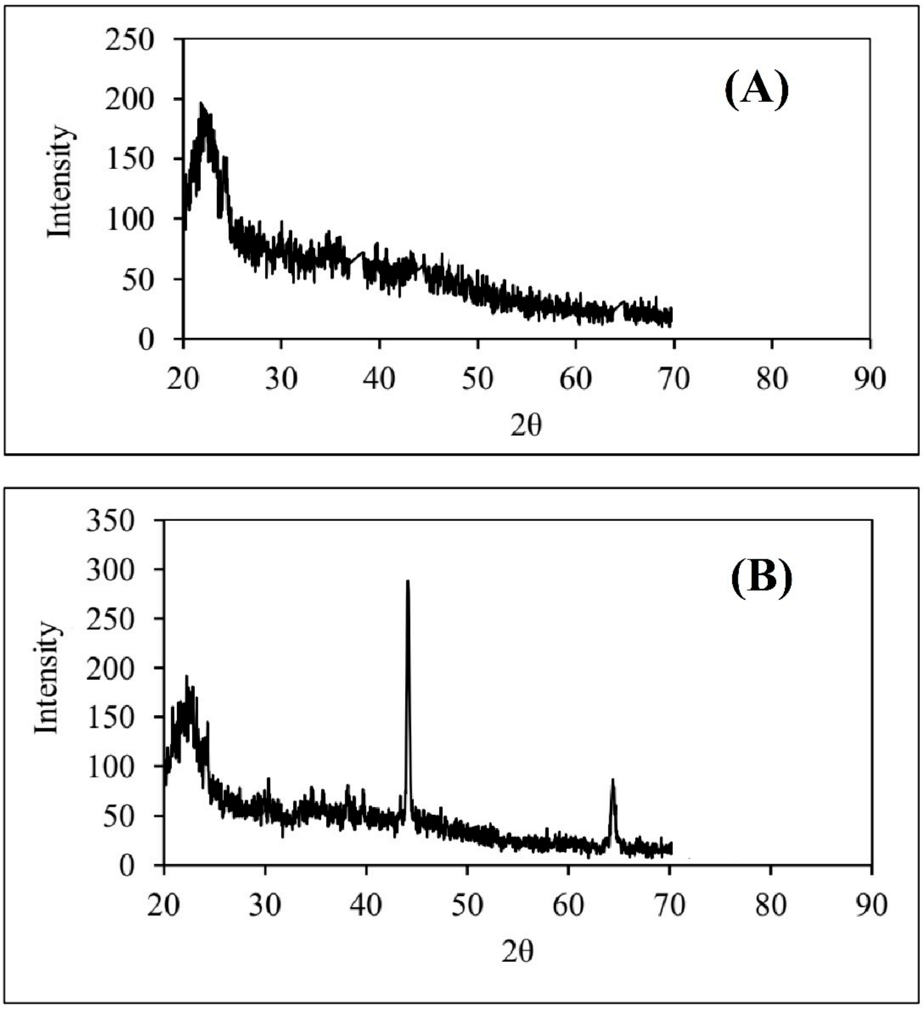

Figure S1. XRD analysis of chemically modified biochar before Cr(VI) adsorption (**A**) and after Cr(VI) adsorption (**B**)

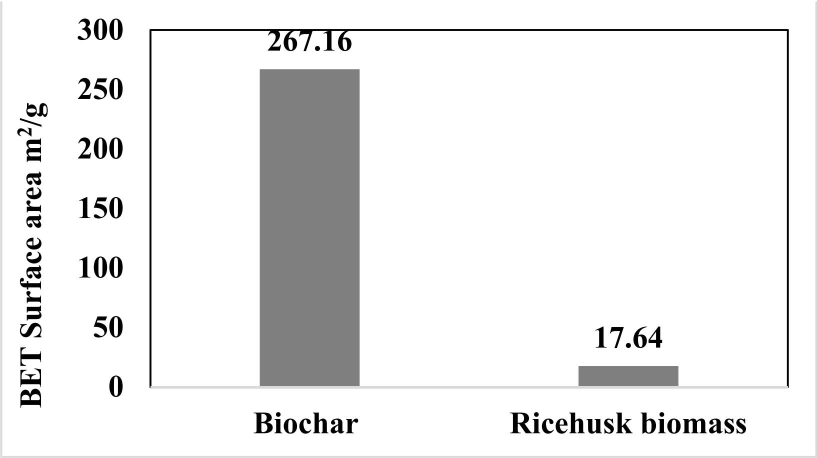

Figure S2. BET surface area of ricehusk biomass and biochar.

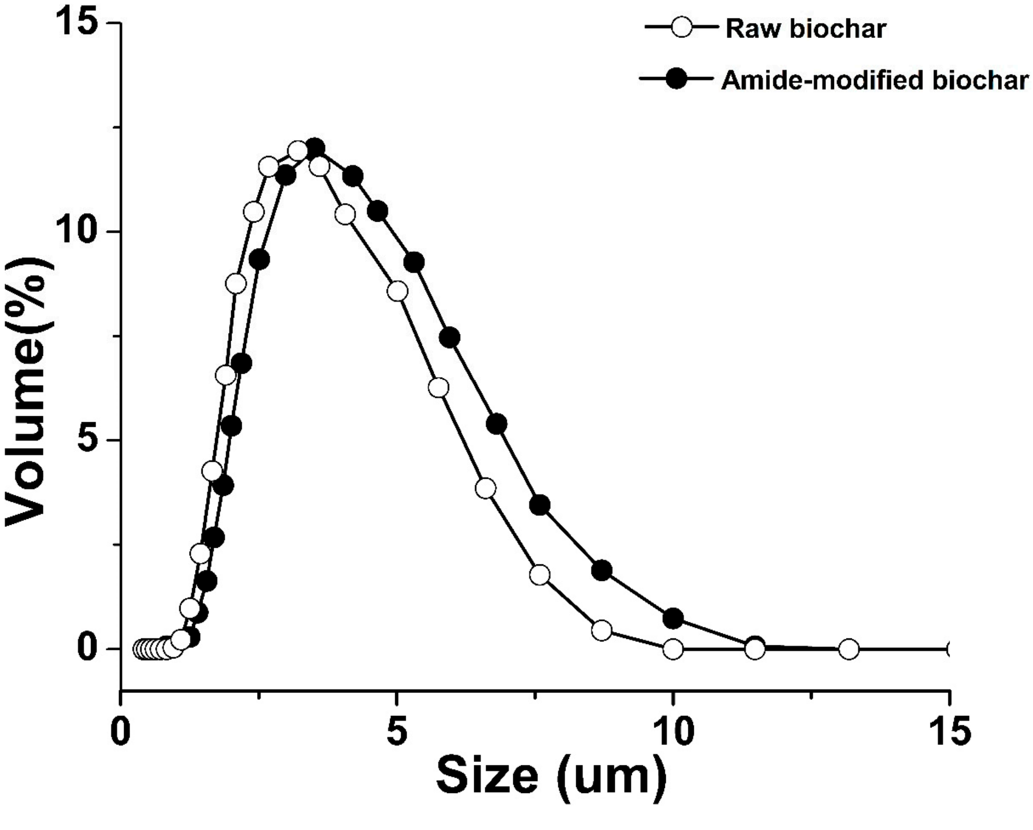

Figure S3. Volume based particle size distribution of raw and chemically modified biochar.

Table S1. Physico-chemical characteristics of industrial wastewater.

**Parameters**	**Values**
pH	7.3 ± 0.4
BOD (mg/l)	897 ± 150
COD (mg/l)	1330 ± 470
TDS (mg/l)	998 ± 223
Conductivity (μS/cm)	2790 ± 330
DCO (mg/l)	549.5 ± 280
DBO_5 (mg/l)	363 ± 210
Hardness	5.92 ± 2.15
Salinity (g/l)	1.3 ± 0.4
